# Isolated obesity resistance condition or associated with aerobic exercise training does not promote cardiac impairment

**DOI:** 10.1590/1414-431X2020e10669

**Published:** 2021-07-16

**Authors:** J.P. Cordeiro, V.L. da Silva, D.H. Campos, A.C. Cicogna, A.S. Leopoldo, A.P. Lima-Leopoldo

**Affiliations:** 1 Programa de Pós-Graduação em Educação Física, Centro de Educação Física e Desportos, Universidade Federal do Espírito Santo, Vitória, ES, Brasil; 2 Departamento de Clínica Médica, Faculdade de Medicina de Botucatu, Universidade Estadual Paulista, Botucatu, SP, Brasil

**Keywords:** High-fat diet, Obesity, Resistance to obesity, Exercise training, Myocardial function, Calcium handling

## Abstract

Mechanisms involved in cardiac function and calcium (Ca^2+^) handling in obese-resistant (OR) rats are still poorly determined. We tested the hypothesis that unsaturated high-fat diet (HFD) promotes myocardial dysfunction in OR rats, which it is related to Ca^2+^ handling. In addition, we questioned whether exercise training (ET) becomes a therapeutic strategy. Male Wistar rats (n=80) were randomized to standard or HFD diets for 20 weeks. The rats were redistributed for the absence or presence of ET and OR: control (C; n=12), control + ET (CET; n=14), obese-resistant (OR; n=9), and obese-resistant + ET (ORET; n=10). Trained rats were subjected to aerobic training protocol with progressive intensity (55-70% of the maximum running speed) and duration (15 to 60 min/day) for 12 weeks. Nutritional, metabolic, and cardiovascular parameters were determined. Cardiac function and Ca^2+^ handling tests were performed in isolated left ventricle (LV) papillary muscle. OR rats showed cardiac atrophy with reduced collagen levels, but there was myocardial dysfunction. ET was efficient in improving most parameters of body composition. However, the mechanical properties and Ca^2+^ handling from isolated papillary muscle were similar among groups. Aerobic ET does not promote morphological and cardiac functional adaptation under the condition of OR.

## Introduction

Obesity is characterized by excess adipose tissue sufficient to cause health damage. Moreover, this condition is a complex multifactorial disease that is influenced by genetic and environmental factors (1), and it is directly linked to the development of risk factors associated with metabolic and inflammatory changes as well as with impairment of cardiac function and morphology (2–4). Thus, the current obesity epidemic is a consequence of an interaction between obesity-predisposing genes and environmental factors that induce the expression of the obese phenotype (5).

A particular aspect of obesity is the different responses to the same high-fat diet. Interestingly, not all animals become obese when consuming a high-fat diet (6). Scientists investigating animals fed on a high-fat diet identified a third category that does not become obese, presenting similar or lower weight gain than animals fed on a low-fat diet (7). This characteristic gives these rats the capacity to resist the morphological condition of obesity, being classified as obese-resistant (OR). OR rats present lower weight gain and body fat deposition compared with obese rats, despite similar food intake (1). Thus, resistance to obesity seems to reflect the ability of these animals to present higher energy expenditure as an adaptive response to high-calorie intake, which compensates weight gain (8).

However, it is unclear whether resistance to obesity has the capacity to maintain the immunity against heart injury caused by obesity (9). Research on animals submitted to a moderate-fat diet found no abnormalities in cardiac function in OR rats. On the other hand, Louis et al. (10) showed that OR rats submitted to high-fat diet presented an increase in myocardial isovolumetric relaxation time, promoting cardiac dysfunction. Several factors have been associated with cardiac impairment in experimental models, including intracellular Ca^2+^ handling, one of the main mechanisms of myocardial contractility and relaxation (11).

Considering the negative impact of inadequate eating habits and sedentary lifestyle on the heart, intervention measures have been studied, including exercise training (ET) (12–14). According to the literature, among its many benefits, this non-pharmacological tool has a cardioprotective effect and reverses cardiac abnormalities (15).

Paulino et al. (16) observed the role of ET as a strategy of improving cardiac function in obesity. Thereby, ET increases the expression of myocardial proteins and improves cardiac function (14). Ca^2+^ handling and the increased sensitivity of myofilaments are possible mechanisms related to the optimization of the cardiac performance after the training period (17,18).

The behavior of the cardiac function in OR rats through an unsaturated high-fat diet is still somewhat obscure as is the action of exercise as a cardioprotective factor in this condition. In addition, there are few studies about Ca^2+^ handling in OR rats and its relationship with ET. Therefore, we investigated the influence of ET on cardiac function and Ca^2+^ handling under the condition of resistance to obesity. The hypothesis was that ET induces a cardioprotection against possible cardiac abnormalities and prevents Ca^2+^ handling impairment.

## Material and Methods

### Animal care

Thirty-day-old male Wistar rats (≌100 g) obtained from the Animal Center of Botucatu Medical School (Brazil) were individually caged. The environment was kept at a constant temperature of 23±3°C and relative humidity of 60±5%. To avoid adverse effects from circadian rhythm alteration (8), considering that rats have nocturnal habits, the room was preserved under a 12 h reversed light/dark cycle that started at 9:00 a.m.

All experiments and procedures were performed in accordance with the Guide for the Care and Use of Laboratory Animals published by the U.S. National Institutes of Health and with the current Brazilian laws. The Ethics Committee of the Federal University of Espírito Santo approved the experimental protocol (No. 1036-2013).

### Experimental protocol

After a period of 7 days for acclimatization, the rats were randomly assigned into two groups: standard diet (SD, n=40) and unsaturated high-fat diet (HFD, n=40). All animals had free access to water and chow (50 g/day). The SD group was fed on a standard diet (RC Focus 1765, Brazil) containing 12.3% of its kilocalories from fat, 57.9% from carbohydrates, and 29.8% from protein. HFD animals were fed on a cycle of four unsaturated high-fat diets (RC Focus 2413, 2414, 2415, and 2416), only differing in their flavoring, but not different in micro- or macronutrients (9). The high-fat diets contained 49.2% of their kcal from fat, 28.9% from carbohydrates, and 21.9% from protein as previously described (11). The standard diet contained soybean oil, whole corn, wheat bran, soybean bran, dicalcium phosphate, sodium chloride, fish and meat flour, antioxidant additive, and a vitamins and minerals mixture. The dietary ingredients used to prepare the high-fat diets were sodium chloride, casein, powdered milk, soybean protein concentrate, whole corn, cracker flour, dicalcium phosphate, Ca2þ carbonate, emulsifier, antioxidants and flavoring (cheese, vanilla, chocolate, and bacon) additives, and a vitamins and minerals mixture. The high-fat diets were calorically rich compared to low-fat diet (high-fat diet=3.65 kcal/g *vs* low-fat diet=2.95 kcal/g), which provided 80 and 20% of the fat-derived calories, respectively (consisting of saturated and unsaturated fatty acids).

**Figure 1:**
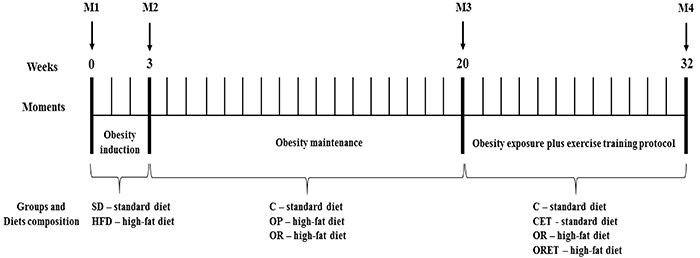
Experimental protocol timeline of 32 weeks. The weeks are represented by individual bars. Initially, animals were distributed into two groups, fed on standard diet (SD) and high-fat diet (HFD). After 3 weeks of experimental protocol (obesity induction), body weight of SD and HFD presented a statistical difference, and this moment was considered the onset of obesity and obesity resistance. The obesity resistance and obesity maintenance period lasted 17 weeks and then the animals were redistributed into two more groups for the practice or not of exercise training, maintaining the same diets and composing the groups: control (C); control submitted to the exercise training protocol (CET); obese-resistant (OR); and obese-resistant submitted to the exercise training protocol (ORET). M1: Beginning of the experimental protocol, induction to obesity; M2: Beginning of obesity; M3: Characterization of obesity prone (OP) and obesity resistance (OR), redistribution of groups to the beginning of the exercise training protocol (CET and ORET); M4: End of the experimental protocol, euthanasia, and start of study analyses.

During the experimental protocol, body weight was recorded weekly. To analyze whether dietary-induced obesity was associated with alterations in nutritional behavior, food consumption (FC) was measured daily. Calorie intake (CI) was calculated weekly by the average weekly FC × dietary energetic density. Feed efficiency (FE), the ability to transform consumed calories into body weight, was determined by the following formula: mean body weight gain (g) / total calorie intake (kcal). The experimental protocol consisted of a total period of 32 weeks, being divided into four moments (Figure 1) and into *Experiment 1* and *Experiment 2*. *Experiment 1* involved the process of dietary submission, and induction and maintenance of obesity (initial week to week 20). *Experiment 2* was started after the 20th week, where the characterization of the experimental groups and the ET protocol were performed until the 32nd week.

### Initial moment of obesity and characterization of OR

The beginning of obesity was determined from the weekly evolution of the body weight of rats. This procedure was used to verify the initial moment and, consequently, the duration of obesity of obesity-prone animals, according to previous studies (19,20). After this moment, SD and HFD rats were maintained on their respective group for 17 consecutive weeks.

**Figure 2:**
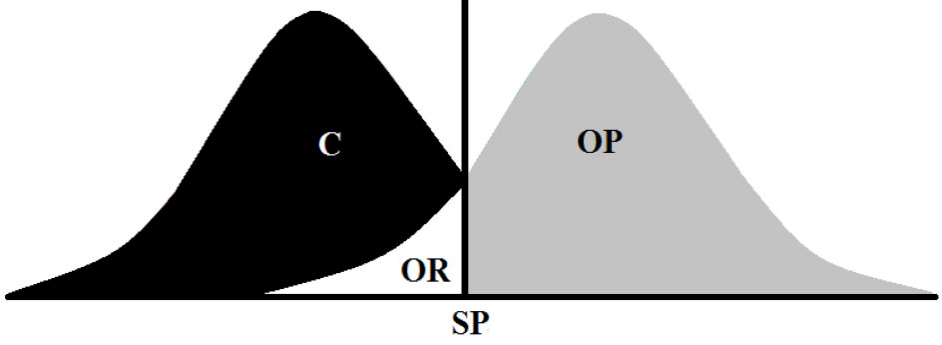
Schematic representation of the criterion used to compose the groups. SP: point of separation (cutoff); (C) composition of the control; OP: obese-prone; OR: obese-resistant.

In the 20th week, a 95% confidence interval (CI) was determined based on the means of body weight from the SD and HFD rats, and was adopted as the cutoff point between the groups, the midpoint between the SD upper limit, and the HFD lower limit. From this point, SD animals with body weight values higher than the cutoff were excluded from the group, and those remaining formed the control group (C). From the HFD group, animals with body weight values higher than the cutoff were classified as obese-prone (OP) and those that were below this point were classified as obese-resistant (OR) (Figure 2). Finally, 27 animals from the C group, 19 characterized as OR, and 21 classified as OP remained in the study.

### Exercise testing and moderate exercise training protocol

After redefining the groups in C (n=26) and OR (n=19), the animals were submitted to the ET protocol. Considering the objective of our study, OP animals remained in the experimental protocol until the 20th week, only aiming at being a parameter for identifying resistance to obesity. Therefore, only the animals of the C and OR groups were submitted to ET, which lasted 12 weeks, constituting the C and OR groups with presence or absence of exercise training (C=12, CET=14, OR=9, and ORET=10).

At week 21 of the experimental protocol, the moderate ET protocol started (Figure 1), adapted from Mostarda et al. (15). The ET (55-70% of the maximum running speed) was performed on a motor treadmill at zero inclination (Insight Equipamentos, Brazil), five days/week during 12 weeks. From weeks 1 to 2, aerobic training was performed with a progressive increase of duration (15 to 30 min/day) and intensity (55% of the maximum running speed). From the 3th and 4th weeks, duration was augmented from 30 to 45 min/day, but the intensity was kept constant (60%). In the 5th and 6th weeks, the rats trained with a progressive increase of duration (45 to 60 min/day) and 65% of intensity. Finally, in the last 6 weeks, the trained rats performed the aerobic exercise training with the same duration of week 6, but the intensity was 70%. All training sessions took place during the afternoon (from 2:00 to 5:00 p.m.). Before starting the aerobic training protocol, the animals were placed on the treadmill for acclimatization and were adapted to the procedure (15 min/day; 5 m/min). Subsequently, the trained groups performed a maximal treadmill test with an initial speed of 9 m/min. Every 3 min, the speed was increased by 3 m/min until animal exhaustion. The test was performed until the rats were unable to maintain the pace of the motor treadmill belt. The tests were performed at baseline (week 1) and in the third, fifth, and seventh weeks of ET to adequate the exercise training intensity (20).

### Nutritional assessment

After starting the experimental protocol, FC, CI, FE, and body weight (BW) were recorded weekly. CI was calculated as follows: CI = average weekly food consumption calorie value of each diet. FE (%) is the ability to convert calorie intake to BW, and it was determined as the mean BW gain (g) / total calorie intake (kcal) × 100. BW, total body fat (BF), and adiposity index (AI) were measured to assess obesity. At the end of the experimental protocol (32 weeks), the animals were anesthetized with an intramuscular injection of ketamine (50 mg/kg) and xylazine (0.5 mg/kg), euthanized, and thoracotomized, and the adipose tissue pads were dissected and weighed. AI was calculated with the following formula: AI = [total body fat (BF) / final BW] × 100. BF (g) was measured based on the sum of the individual fat pad weights as follows: BF = epididymal fat + retroperitoneal fat + visceral fat.

### Comorbidities and hormones

ET presents beneficial effects on comorbidities associated with excess of adipose tissue. Thus, at the end of the experimental protocol, systolic blood pressure (SBP), glucose tolerance, homeostatic model assessment index (HOMA-IR), lipid profile, and blood levels of leptin and insulin were assessed, as previously described (9).

### Morphological analysis

After euthanasia, the heart, ventricles, and tibia were separated, dissected, weighed, and measured. Cardiac remodeling at the macroscopic level, through which we can identify the presence or absence of hypertrophy, was determined by analyzing the following parameters: heart weight (HW), weights of left and right ventricles (LV and RV), and their relation to tibia length (3).

### Myocardial function

Myocardial contractility was evaluated by studying isolated LV papillary muscle, as previously described (9). The following mechanical parameters were measured from isometric contraction: maximum developed tension (DT [g/mm^2^]), positive tension derivative (+dT/dt [g/mm^2^/s]), and negative tension derivative (-dT/dt [g/mm^2^/s]). The mechanical behavior of the papillary muscle was assessed under baseline condition at 2.5 mM Ca^2^ and after the following inotropic and lusitropic maneuvers: increases in extracellular Ca^2+^ concentration (to test their effects on myofilament machinery) and post-rest contraction (PRC), mainly related to sarcoplasmic reticulum storage and release capacity. All variables were normalized per cross-sectional area of papillary muscle (CSA).

### Histological study

LV transverse sections of animals from each group were fixed in 10% buffered formalin and embedded in paraffin. Histological sections of 4-μm thickness were stained with a hematoxylin-eosin (HE) solution and designed with a 40-fold increase with the aid of a microscope (LeicaMikroshopie & System GmbH, Germany) coupled with a video camera, which sends digital images to a computer with an image analysis program (ImagePro-plus, Media Cybernetics, USA). For calculating the sectional areas of myocytes, about 50 to 70 cells were measured.

Interstitial collagen fraction (CF) was determined for the entire picrosirius red-stained cardiac section using an automatic image analyzer (Image-Pro Plus 3.0). The components of the cardiac tissue were identified according to color level as follows: red for collagen fibers; yellow for myocytes; and white for interstitial space. CF was calculated as the sum of all connective tissue areas divided by the sum of all connective tissues and myocyte areas. The quantification of the interstitial collagen fraction was performed using from 30 to 40 fields by fragment. Perivascular collagen was excluded from this analysis.

### Statistical analysis

Data on general characteristics, comorbidities, cardiac remodeling, and myocardial function are reported as means±SD. Comparisons between groups were performed by two-way analysis of variance (ANOVA) for independent samples, followed by Bonferroni's *post hoc* test. Two-way repeated measures ANOVA was used to evaluate positive and negative inotropic effects on myocardial function. The level of significance was set at 5%.

## Results

### Experiment 1

**Figure 3:**
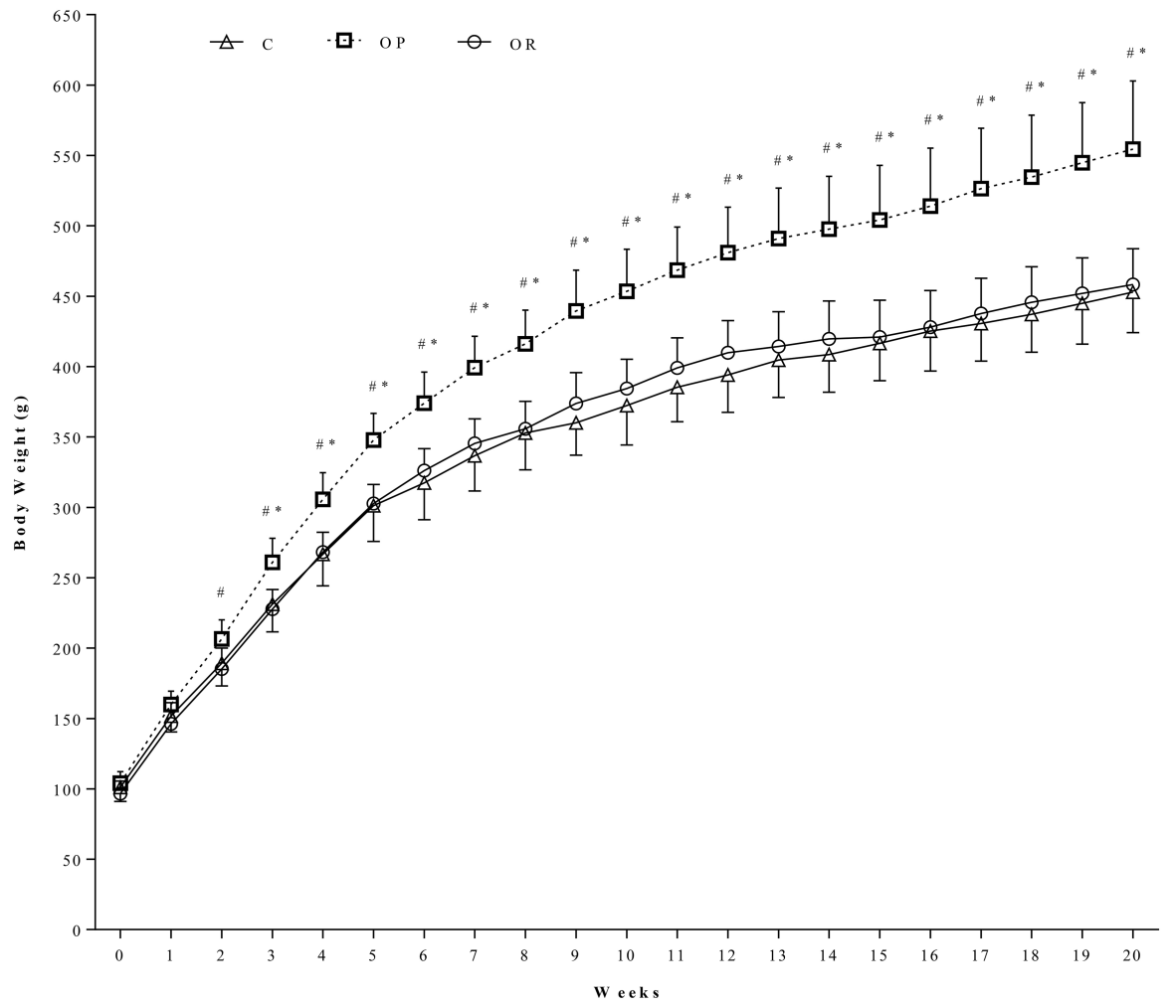
Evolution of body weight during the protocol of induction and exposure to obesity after 20 weeks of treatment. Control (C, n=27), obese-resistant (OR, n=19), and obese-prone (OP, n=21). Data are reported as means±SD. *P<0.05 OP *vs* C; ^#^P<0.05 OR *vs* OP (repeated measures two-way ANOVA followed by Bonferroni's *post hoc* test).

*Characterization of obesity resistance*. Figure 3 shows the evolution of body weight of rats. The OP group featured high values of body weight in relation to the C and OR animals, which values did not differ among them. This moment was regarded as the beginning of obesity (week 3 in this study). At the 20th week, after applying the cutoff point, the redefinition of the groups indicated that within the group exposed to the HFD, 19 animals were characterized as OR and 21 as OP. In addition, 13 animals that presented body weight values higher than the cutoff were excluded from the SD group. In the initial moment, the experimental groups were C (n=27), OP (n=21), and OR (n=19).

Table 1 shows the nutritional characteristics of the groups during the induction protocol and exposure to obesity after 20 weeks. The OP presented higher body weight gain and calorie intake compared with the C and OR. Food consumption has significantly increased in the OP group in relation to the OR, but there was no difference in this parameter between the C and OP groups. In addition, the OR group presented higher calorie intake with a reduced food efficiency in relation to the C and OP.

**Table 1:** Nutritional characteristics during protocol of obesity induction and maintenance after 20 weeks of treatment.

Variables	C (n=27)	OR (n=19)	OP (n=21)
Weight gain (g)	351±28	362±26	450±47*^#^
Food consumption (g/day)	22.3±1.4	20.9±2.8	22.8±0.4^#^
Calorie intake (Kcal/day)	65.9±4.1	76.3±10.2^&^	83.1±6.4*^#^
Food efficiency (%)	3.81±0.23	3.42±0.36^&^	3.87±0.20^#^

Data are reported as means±SD. ^&^P<0.05 OR *vs* C; *P<0.05 OP *vs* C; ^#^P<0.05 OP *vs* OR (ANOVA followed by Bonferroni’s *post hoc* test). C: control; OR: obese-resistant; OP: obese-prone.

### Experiment 2

*Influence of aerobic ET on obesity resistance.* The OP group was used in our study only to identify and characterize animals that present resistance to obesity. The C and OR groups were redistributed into two groups, regarding the presence or absence of aerobic ET.

One animal of the control group died. Thus, in the second stage of the experimental protocol (from the 21st to the 32nd week), the study was composed of four groups: control (C; n=12), control submitted to ET (CET; n=14), obese-resistant (OR; n=9), and obese-resistant submitted to ET (ORET; n=10).

There were no differences in BW among groups. Food consumption of the OR and ORET rats was lower than the C and CET groups, respectively (Table 2). In addition, the calorie intake was higher only in the OR group compared with the C group. The OR and ORET groups featured greater values of epididymal fat pad compared with their respective control groups. There was no significant difference for the other variables under HFD effect. Moreover, the ET groups (CET and ORET) did not show changes in food consumption and calorie intake. However, ET was efficient in reducing the final body weight, weight gain, and retroperitoneal, visceral, epididymal, and body fats as well as food efficiency and adiposity index in relation to groups with no exercise (C and OR), respectively.

**Table 2:** Nutritional characteristics after the aerobic exercise training protocol.

Variables	C (n=12)	CET (n=14)	OR (n=9)	ORET (n=10)
Food consumption (g/day)	22.5±0.62	22.7±0.57	20.2±0.71*	19.0±0.68^£^
Calorie intake (Kcal/day)	66.5±2.10	67.0±1.95	73.6±2.43*	69.4±2.30
Food efficiency (%)	0.93±0.10	0.41±0.10^&^	0.90±0.12	0.48±0.11^#^
IBW (g)	452±8.05	453±7.46	463±9.30	454±8.82
FBW (g)	509±9.25	479±8.56^&^	523±10.68	486±10.1^#^
Weight gain (g)	56.4±6.86	26.5±6.36^&^	59.8±7.93	31.9±7.52^#^
Epididymal fat (g)	10.1±0.83	5.84±0.77^&^	13.0±0.96*	9.05±0.91^#£^
Retroperitoneal fat (g)	15.6±1.28	10.6±1.20^&^	17.8±1.48	11.1±1.40^#^
Visceral fat (g)	6.91±0.51	5.13± 0.47^&^	7.03±0.58	4.76±0.55^#^
Body fat (g)	32.6±2.37	21.5±2.19^&^	37.8±2.73	24.9±2.59^#^
Adiposity index (%)	6.35±0.42	4.46±0.38^&^	7.22±0.48	5.09±0.45^#^

Data are reported as means±SD. *P<0.05 OR *vs* C; ^#^P<0.05 ORET *vs* OR; ^&^P<0.05 CET *vs* C; ^£^P<0.05 ORET *vs* CET (two-way ANOVA followed by Bonferroni's *post hoc* test). IBW: initial body weight at week 21; FBW: final body weight at week 32; body fat: sum of fat pads; C: control; CET: control submitted to exercise training; OR: obese-resistant; ORET: obese-resistant submitted to exercise training.

**Figure 4:**
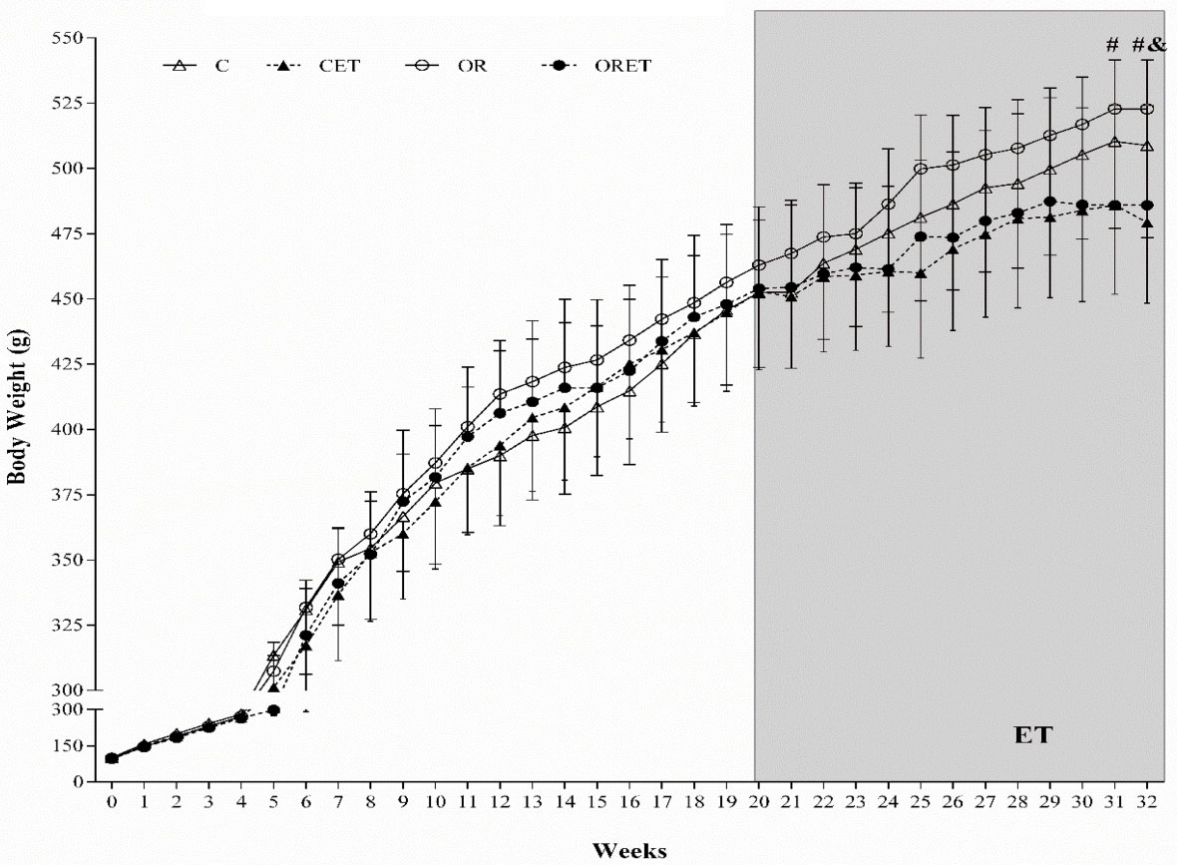
Evolution of body weight during the period of exposure to diet and aerobic exercise training (ET) protocol. Control group (C, n=12), exercised control (CET, n=14), obese-resistant (OR, n=9), and obese-resistant exercised (ORET, n=10). Data are reported as means±SD. ^#^P<0.05 ORET *vs* OR; ^&^P<0.05 CET *vs* C (repeated measures two-way ANOVA followed by Bonferroni's *post hoc* test).

Considering the BW evolution among the OR groups, there was a reduction in the ORET's BW value compared to OR at weeks 31 and 32 (Figure 4). In the C groups, ET promoted reduction in BW only at the 32nd week (CET < C).

The HFD promoted higher low-density lipoprotein (LDL) values in the OR groups (OR *vs* C and ORET *vs* CET; P<0.05). In addition, the insulin levels were lower in the OR compared to the C group. However, the presence of aerobic ET resulted in a decrease in LDL levels in the CET and ORET compared with C and OR, respectively. SBP was higher in the ORET than in the OR and CET groups. Both high-fat diet and aerobic exercise training did not promote changes in glucose, total cholesterol, triglycerides, protein, HDL, lactate, HOMA-IR, and leptin levels among groups. In addition, the OR groups promoted higher area under the curve for glucose (AUC) than the C groups (OR *vs* C and ORET *vs* CET, P<0.05) (Table 3).

**Table 3:** Biochemical, hormonal, glycemic, and hemodynamic profiles.

Variables	C (n=12)	CET (n=14)	OR (n=9)	ORET (n=10)
Total cholesterol (mg/dL)	78.4±5.39	76.6±4.99	78.4±6.22	68.5±5.90
HDL (mg/dL)	24.5±1.39	27.1±1.29	26.1±1.61	24.1±1.53
LDL (mg/dL)	10.9±1.34	7.39±1.05^&^	15.2±1.31*	10.8±1.24^#£^
Total proteins (mg/dL)	6.21±0.11	5.94±0.10	6.03±0.12	5.70±0.12
Triglycerides (mg/dL)	52.5±4.35	59.1±4.03	51.9±5.03	46.8±4.77
Glucose (mg/dL)	144±9.21	146±8.53	161±10.6	144±10.1
SBP (mmHg)	109±3.50	112±3.24	99.5±4.04	125±3.83^#£^
Lactate (mg/dL)	14.7±0.74	14.2±069	13.7±0.86	14.2±0.81
HOMA-IR	11.9±1.23	8.57±1.23	7.81±1.41	8.10±1.34
Leptin (ng/mL)	1.09±0.37	0.8±0.48	1.11±0.44	0.90±0.44
Insulin (ng/mL)	0.92±0.46	0.75±0.43	0.56±0.24*	0.56±0.34
AUC (mg/dL/min)	19041.9±779.4	19670.9±721.6	21959.2±900.0*	22560.0±853.8^£^

Data are reported as means±SD. *P<0.05 OR *vs* C; ^£^P<0.05 ORET *vs* CET; ^#^P<0.05 ORET *vs* OR; ^&^P<0.05 CET *vs* C (two-way ANOVA followed by Bonferroni's *post hoc* test). C: control; CET: control submitted to exercise training; OR: obese-resistant; ORET: obese-resistant submitted to exercise training; HDL: high density lipoprotein; LDL: low density lipoprotein; SBP: systolic blood pressure; AUC: area under the curve for glucose.

**Figure 5:**
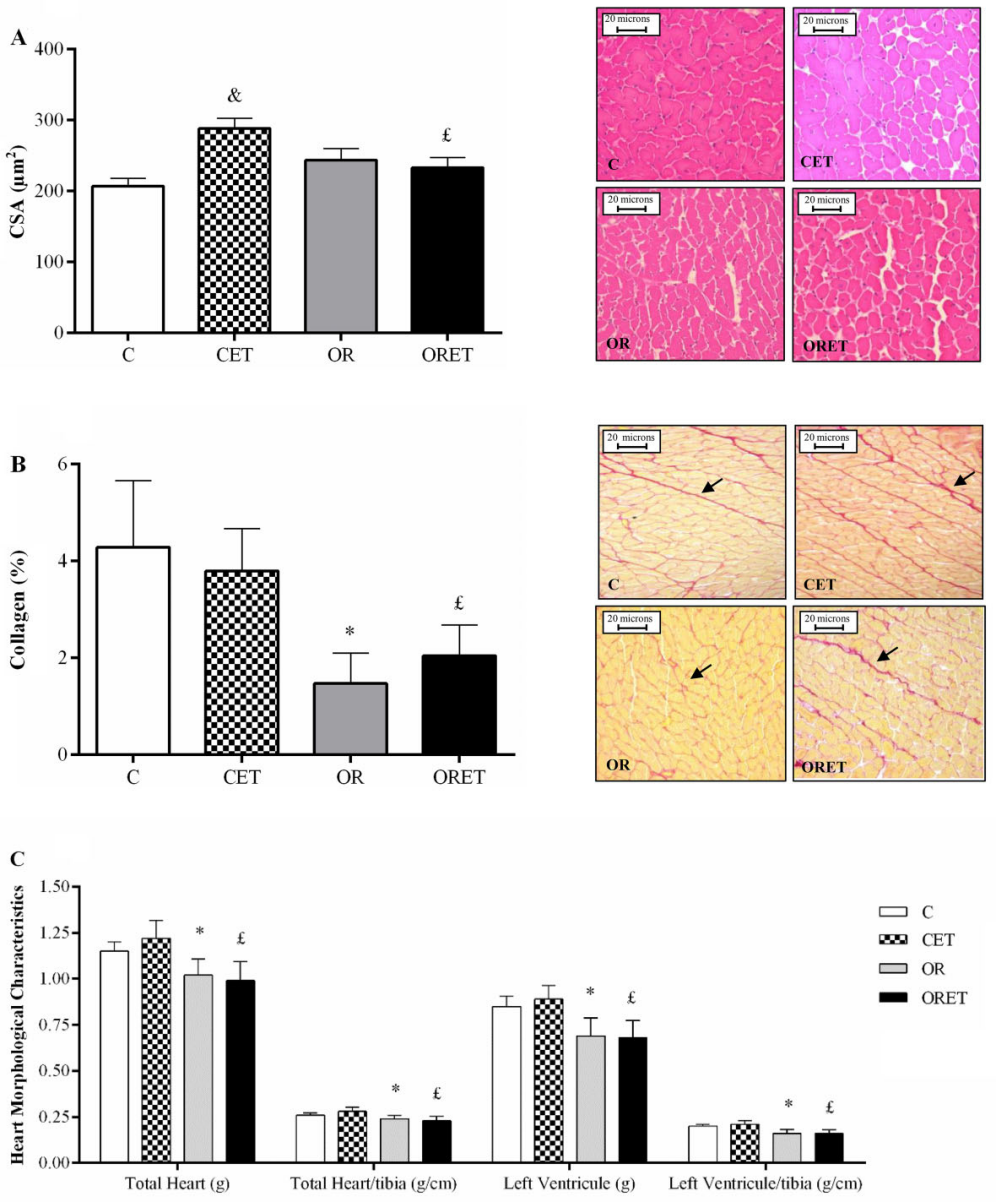
A, Cross-sectional area (CSA) performed on left ventricle (LV) fragment. **B**, Collagen deposition in LV fragment (scale bars 20 μm). **C**, Cardiac and tibia morphological characteristics. Control group (C, n=12), exercised control (CET, n=14), obese-resistant (OR, n=9), and obese-resistant exercised (ORET, n=10). Data are reported as means±SD. *P<0.05 OR *vs* C; ^£^P<0.05 ORET *vs* CET; ^&^P<0.05 CET *vs* C (two-way ANOVA followed by Bonferroni's *post hoc* test).

Aerobic ET promoted a significant increase in CSA in the CET when compared with the C group (Figure 5A). Furthermore, CSA was reduced in the ORET group in relation to the CET. There was no significant difference between the other groups (C *vs* OR and ORET *vs* OR). The OR animals featured lower deposition of collagen in relation to their respective controls (ORET *vs* CET and OR *vs* C; P<0.05) (Figure 5B). On the other hand, ET did not promote significant changes between groups in this parameter. Total heart, heart to tibia length ratio, LV, and LV to tibia length ratio presented lower values in the OR and ORET groups than in the C and CET groups, respectively (Figure 5C). Tibia length values were similar among groups.

**Figure 6:**
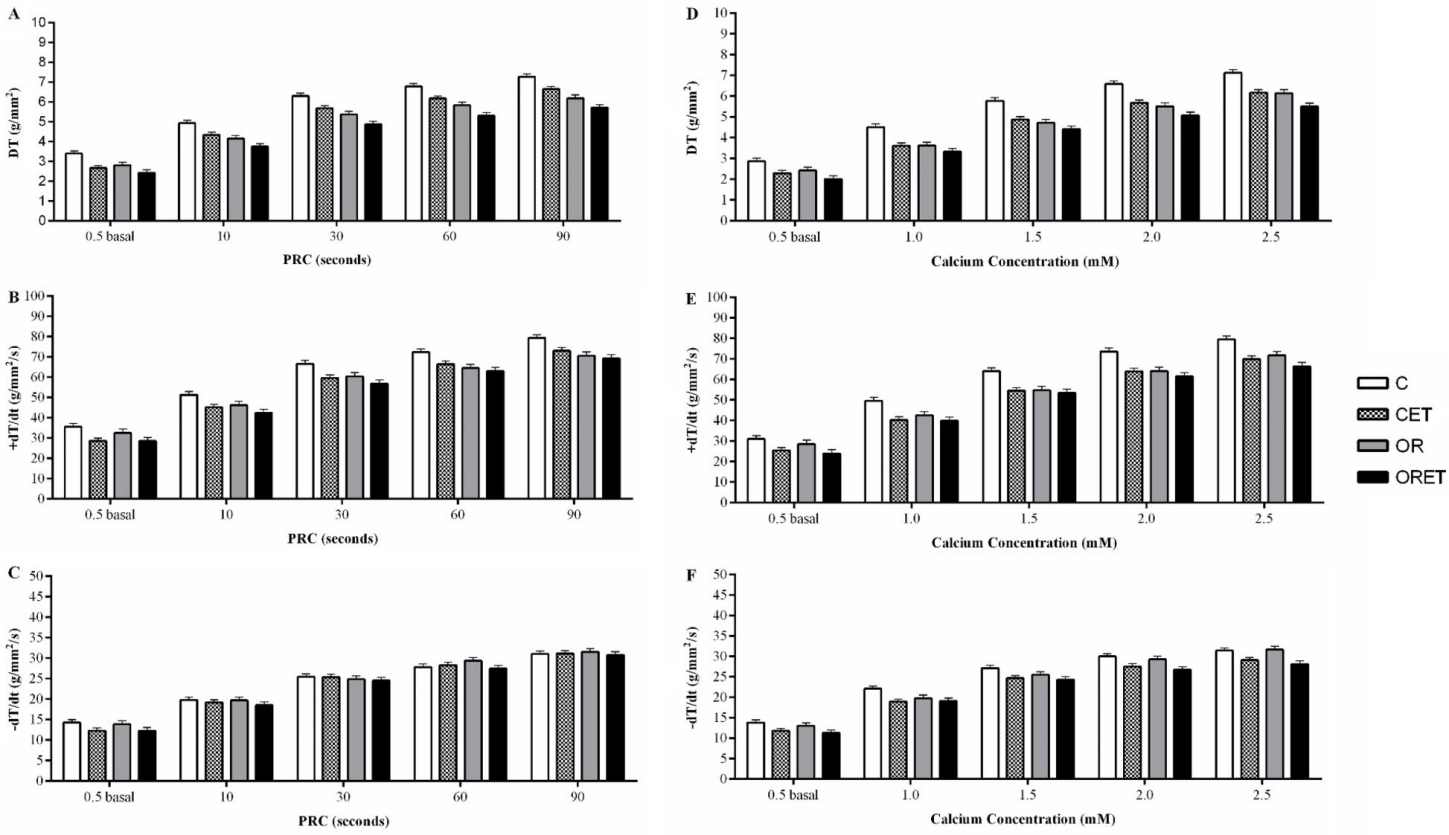
Mechanical properties of isolated papillary muscle from groups. **A**, **B**, and **C**, Post-rest contraction (PRC) and **D**, **E**, and **F,** elevation of extracellular Ca^2+^ concentration (0.5 to 2.5 mM). Control group (C, n=12), control submitted to exercise training (CET, n=14), obese-resistant (OR, n=9), and obese-resistant submitted to exercise training (ORET, n=10). DT: maximum developed tension; +dT/dt: maximum rate of variation of the developed tension; -dT/dt: maximum rate of variation of the decrease in the developed tension. Data are reported as means±SD. There were no statistically significant differences among groups (two-way repeated measures ANOVA followed by Bonferroni's *post hoc* test).

Figure 6A-F summarizes the mechanical properties of isolated papillary muscle from the groups. PRC after 10, 30, 60, and 90 s did not differ among groups for all variables (Figure 6A-C). In addition, the effects of extracellular Ca^2+^ increase on papillary muscle function demonstrated similar results in all variables among the groups (Figure 6D�F).

## Discussion

This study aimed at the development and characterization of a resistance to obesity experimental model in rodents, which confers to these rats the capacity to resist the morphological condition of obesity. The main findings of current study were that the condition of resistance to obesity presented adiposity similar to the control group without cardiac damage. In addition, the ET did not promote effects on cardiac function and Ca^2+^ handling.

According to Levin et al. (7), resistance to obesity is associated with the complex interaction between various metabolic and environmental factors. Thus, animals with resistance to obesity have the ability to maintain low weight gain and body fat deposition even when eating high-calorie diets (6,7). It is noteworthy that studies whose authors use the OR identification of animals by submission to the diet present a greater approximation of the characteristics observed in humans (8), considering that lean phenotypes are observed in some individuals with different dietary patterns and level of physical activity (6).

In our study, OP animals presented high values of BW, while the C and OR rats BWs were similar. This characteristic remained over time for the C, OP, and OR groups during the 32 weeks (Figure 3). Although OR characterization by weight evolution is widely used and has good application, it is not a reliable indication of adiposity, since weight evaluation does not discriminate body components such as muscle mass, bone mass, and fat mass. Weighing does not elucidate the actual ability to convert ingested food into body fat deposits. In addition, the OR group consumed the lowest amount of food in relation to the other groups, however, these animals featured higher daily calorie intake than the C animals (Table 1); the food efficiency of OR rats was reduced compared with the other groups, even with the animals that ingested the SD. This characteristic gives these rats an ability to resist the morphological condition of obesity.

The lower metabolic capacity of OR animals to convert ingested food into weight was confirmed in our study by the lower final BW. In addition, these animals were able to keep the main factors related to the accumulation of adipose tissue reduced, such as smaller deposits of retroperitoneal fat, visceral fat, and sum of fat deposits, as well as the adiposity index compared with the OP group. Akieda-Asai et al. (1), when analyzing the adipocyte sectional area of white adipose tissue, observed that OR animals presented a similar area to the control group and significantly lower area than that of the OP animals; in addition, they featured greater carnitine palmitoyl transferase expression and lesser fatty acid synthase, which corroborates the idea that OR animals have lower fat deposition due to higher lipid oxidation and lower lipogenesis.

Obesity has been associated with several comorbidities, such as glucose intolerance, hyperinsulinemia, insulin resistance, dyslipidemia, and hypertension (7,8). However, in the OR condition there were no significant differences in the serum values of proteins, triglycerides, total cholesterol, and HDL. We emphasize that the HFD promoted higher values of LDL in the OR group, suggesting a risk factor to the cardiovascular system, related to developing comorbidities such as hypertension, type 2 diabetes mellitus, and atherosclerosis.

Another important aspect is that the white adipose tissue not only has the energy storage function, but is also an endocrine organ secreting several hormones (21). The OR group did not develop hyperleptinemia, which can be associated with the low values of the fat deposits, that consequently led to the normal levels of hormone production in this group. Furthermore, although hyperinsulinemia was not observed, a higher AUC was visualized in the OR, characterizing intolerance to glucose. This is an unusual finding, since this characteristic is rarely observed in OR animals in other studies (22). However, few authors have evaluated the behavior of this model exposed to the high-fat diet for a long period, and this response can be caused by time (5).

The relationship between obesity and cardiac morphology is strongly reported in literature, which shows that the increase in the number of adipocytes leads to increased metabolic demand and consequent expansion of circulating volume (4). The subsequent increase in the LV systolic volume induces an increase in cardiac output, which in turn results in systemic arterial hypertension.

Unlike animals that develop obesity, we showed that OR animals submitted to the HFD presented cardiac morphological changes with lower values of total heart and LV sizes, and their respective relationships with the tibia length. These findings could indicate a possible prevention of cardiac hypertrophy (23), since cardiomyocytes CSA was similar to the C group. Furthermore, the lower deposition of collagen observed in the OR group could justify the lower weight of the cardiac chambers (24). In addition, arterial hypertension was not observed in the OR condition, corroborating the findings of Carroll et al. (25).

Alterations in the morphology of the heart can lead to functional impairment (4,26); however, few studies have identified the cardiac characteristics of OR rats, and there is limited information and controversies about the relationship between resistance to obesity and myocardial Ca^2+^ handling (10,25,26). In the current study it was not possible to identify impairments in the cardiac function after performing inotropic and lusitropic maneuvers in isolated papillary muscle preparations from OR rats after 32 weeks of HFD exposure. Therefore, the lack of cardiac damage observed in the OR condition could be due to three central factors: firstly, the intermediate time of exposure to the diet, as reported by Leopoldo et al. (3) and Rider et al. (27); secondly, the absence of excessive accumulation of adipose tissue in OR animals, which in turn causes cardiac damage (27); and thirdly, the HFD composition, which is predominantly composed of unsaturated fatty acids. In this sense, studies (6,10,25) on OR by HFD do not indicate the components and characteristics of fatty acids present in the diet. Thus, due to the reduced number of studies and the divergence of results on the relationship between OR and cardiac function, the mechanisms involved in cardiac dysfunction are unknown in this animal model, making the findings inconclusive.

Several researchers have demonstrated the efficacy of aerobic ET in the reduction of fat percentage and, consequently, of body fat deposits (28). The OR animal presents a metabolic capacity favorable to the use of body energy reserves, even in sedentary conditions. However, little is known about the effects of ET and its comparison with sedentary lifestyle in the OR model.

We showed that ET was efficient in adiposity control by decreasing the weight gain and the final body weight. In addition, dietary efficiency, adiposity index, deposits of retroperitoneal fat, visceral, epididymal and total fat were decreased in the trained groups in relation to the sedentary ones (CET < C and ORET < OR). It is noteworthy that resistance to obesity alone provides the capacity to maintain adiposity at the level of control animals. There was no disparity in food and calorie intake between trained and sedentary groups. Levin and Dunn-Meynell (29) reported that the ET factor seems to promote appetite control and consequently regulates calorie intake, a result we did not observe in our study. Thus, the benefits observed in the improvement in body composition, evidenced in our study, are directly related to the ET effect.

Although the ET promoted beneficial effects on adiposity, this non-pharmacological intervention caused alterations only in LDL levels, but it was unable to reverse the glucose intolerance on OR condition. Thus, ET seems to be a protective factor against coronary heart disease in OR animals, mainly because it reduces the risk of atheroma plaque formation due to the decrease in circulating LDL, even without changes in serum HDL concentrations (30). In addition, the lack of a positive response of the ET effect on glucose intolerance contradicts the results reported in the literature, which has shown that aerobic physical training attenuates glucose intolerance (31).

Many factors are associated directly and indirectly with genesis and progression of hypertension, such as metabolic diseases (diabetes mellitus and obesity) (21). Hypertension is characterized by cardiac hypertrophy and dysfunction and elevated levels of blood pressure (BP) and sympathetic tone (14,21). It is well known that the rostral ventrolateral medulla is a main active region for central regulation of the cardiovascular function and plays a key role in maintaining resting BP and sympathetic tone (21). In our study, the SBP of the ORET group was significantly higher than that of the sedentary OR as well as the CET. These findings contradict the literature that suggest that aerobic ET may contribute to reductions of proinflammatory cytokines, reactive oxygen species, and regulatory mechanisms of the positive feedback cycle involved with hypertension and cardiac hypertrophy. The evidence provides insight for the beneficial effect of ET on hypertension and cardiac hypertrophy (3,14,15). However, the result of our study points to an interaction effect between HFD and ET, since this condition was not observed in the exercised group submitted to SD. It is worth noting that the few researchers who evaluated the relationship between physical activity and obesity-resistance did not evaluate blood pressure (32–37).

In relation to the main objective of study, the findings showed that aerobic ET did not promote cardiac morphological and functional alterations, as well as in the Ca^2+^ handling in OR condition. In addition, decreased weight of cardiac chambers and reduced collagen deposition did not seem to interfere with the heart physiology on resistance to obesity, since the cross-sectional area of cardiomyocytes, water accumulation in tissues, and cardiac function assessed in the isolated papillary muscle remained unchanged in this condition. Importantly, aerobic ET attenuates the tumor growth factor (TGF)-β positive stimulation and downstream effectors of fibrosis in the rat heart (37). Poret et al. (38), evaluating cardio-metabolic risk factors in obesity-prone, Osborne-Mendel, and obesity-resistant S5B rats, showed that the LV weight and wall thickness were decreased in S5B rats with consumption of HFD. Furthermore, the authors observed that brain natriuretic peptide (BNP) and connective tissue growth factor (CTGF) mRNA expressions in LV were lower in S5B rats. BNP is a peptide hormone released from LV in response to volume and pressure overload, being described as a marker of cardiac health and one of the most relevant markers of cardiac hypertrophy. Another explanation for reduced collagen could be related to CTGF gene expression, which is associated with the increased potential for fibrosis (38). The absence of cardiac remodeling in our results could be related to molecular factors; however, these were not evaluated. We did not find studies about the influence of chronic ET on cardiac parameters in the condition of resistance to obesity.

Despite literature showing that OR rats exhibit cardiovascular risk factors (37), several agents such as accumulation of interstitial collagen (10) and ultrastructural damage (11) have been proposed as contributing to cardiac dysfunction in different models of heart disease (3). However, one of the most important regulatory mechanisms of myocardial contraction and relaxation (Ca^2+^ handling and cardiac function) remained unchanged in this condition, even with the addition of ET.

Cardiometabolic risk factors are differentially regulated by the susceptibility to develop obesity. Metabolic risk factors (inflammation, angiogenesis, oxidative stress) were more prevalent in the obese rats, compared to OR rats. Whereas cardiovascular key biomarkers were elevated in the obesity condition, these data suggested that an increased susceptibility to develop obesity is linked to an increased risk for cardio-metabolic disease (38,39). In this context, the OR group presented lower insulin value and larger area under the glycemic curve. Perhaps, glucose metabolism is the key to explain alterations of cardiac function, since glucose metabolism was the only one alteration observed in this model (6).

Alterations of glucose metabolism may lead to reduction of cardiac contraction strength, indicated by our results showing both contraction and relaxation of papillary muscles were slowed down. In this condition, damage was described on the transient Ca^2+^, decreased number of ryanodine receptors, and decreased mRNA and protein levels of SERCA2 and NCX exchanger. Significant alterations were also described in contractile proteins.

Curiously, the AUC was higher in fat-fed rats, independent of weight gain. High-fat feeding *per se* induces insulin resistance and it may inhibit skeletal muscle cellular fatty acid oxidation via reduced AMP protein kinase activity and reduced Glut4 mRNA and protein content. Saturated fats, in particular, may impair muscle insulin sensitivity because of storage of lipid metabolites other than triglycerides (39). However, although the OR animals presented altered values of insulin and area under the glycemic curve, these animals did not present higher fasting glucose values that could characterize diabetes. Thus, OR animals appear to maintain the metabolic functions preserved by the higher efficiency of lipid oxidation systems and this seems to promote cardiac protection. This characteristic corroborated the findings by Carrol et al. (25) that analyzed the cardiac adaptations by echocardiography in animals submitted to HFD.

Pathological morphological adaptations are also associated in decreased cardiac function. Decrease in diastolic compliance could be associated with collagen deposition (2), and higher collagen concentration is correlated to abnormalities in insulin metabolism. Insulin growth factor induces transforming growth factor beta-1, which directly stimulates collagen expression (35). The findings of the present study are in agreement with the above, as animals from the OR group presented lower values of insulin and interstitial collagen.

In the Benito et al. (40) study, using rats trained with high-intensity aerobic exercise, the animals developed concentric LV hypertrophy at 8 weeks, manifested by increased LV wall thickness and by the interventricular septum ratio for LV diameter at the end of the diastole, progressing to hypertrophy eccentric/ventricular dilation at 16 weeks. In the present study, ET was used as a non-pharmacological intervention with the aim to attenuate possible abnormalities caused by obesity and/or resistance to obesity. However, OP and OR conditions did not promote abnormalities, as expected, and physiological hypertrophy caused by physical exercise was also not identified. This absence of physiological adaptations by ET can be attributed both to the duration of the training protocol adopted here (12 weeks) and to the moderate intensity of the routine, unlike the high intensity used by other authors (40).

In summary, the rats with resistance to obesity, even when subjected to prolonged periods of unsaturated high-fat diet, maintained most of the body composition characteristics similar to the control group and did not have cardiac functional impairment and alterations in Ca^2+^ handling. In addition, moderate intensity aerobic ET did not promote changes in either morphology or cardiac function for OR animals.

### Limitation of the study

Although it is very important to demonstrate the effect of physical exercise in different conditions and/or treatments, one limitation of our study could be regarding the absence of data related to the aerobic capacity and the load adjustment in trained rats (CET and ORET). Possibly, the ideal strategy would be to include the maximal treadmill test throughout the experimental protocol to evaluate the functional capacity/physical conditioning without underestimating the load level of the exercise.
